# ATPsite: sequence-based prediction of ATP-binding residues

**DOI:** 10.1186/1477-5956-9-S1-S4

**Published:** 2011-10-14

**Authors:** Ke Chen, Marcin J Mizianty, Lukasz Kurgan

**Affiliations:** 1 Department of Electrical and Computer Engineering, University of Alberta, Edmonton, AB, Canada

## Abstract

**Background:**

ATP is a ubiquitous nucleotide that provides energy for cellular activities, catalyzes chemical reactions, and is involved in cellular signalling. The knowledge of the ATP-protein interactions helps with annotation of protein functions and finds applications in drug design. The sequence to structure annotation gap motivates development of high-throughput sequence-based predictors of the ATP-binding residues. Moreover, our empirical tests show that the only existing predictor, ATPint, is characterized by relatively low predictive quality.

**Methods:**

We propose a novel, high-throughput machine learning-based predictor, ATPsite, which identifies ATP-binding residues from protein sequences. Our predictor utilizes Support Vector Machine classifier and a comprehensive set of input features that are based on the sequence, evolutionary profiles, and the sequence-predicted structural descriptors including secondary structure, solvent accessibility, and dihedral angles.

**Results:**

The ATPsite achieves significantly higher Mathews Correlation Coefficient (MCC) and Area Under the ROC Curve (AUC) values when compared with the existing methods including the ATPint, conservation-based rate4site, and alignment-based BLAST predictors. We also assessed the effectiveness of individual input types. The PSSM profile, the conservation scores, and certain features based on amino acid groups are shown to be more effective in predicting the ATP-binding residues than the remaining feature groups.

**Conclusions:**

Statistical tests show that ATPsite significantly outperforms existing solutions. The consensus of the ATPsite with the sequence-alignment based predictor is shown to give further improvements.

## Background

Adenosine-5'-triphosphate (ATP) is a multi-functional nucleotide that plays an important role in energy metabolism, signaling, and replication and transcription of DNA. As of July 2010, 3860 structures in the Protein Data Bank (PDB) [1], which constitute about 6% of known protein structures, are annotated as ATP binding. The ATP binding sites are regarded as valuable drug targets for antibacterial and anti-cancer chemotherapy [2,3]. Therefore, the protein-ATP interactions are of significant interest.

Past two decades observed a substantial effort in identification of conserved characteristics of the ATP-binding sites. Most of these approaches are based on a relatively simple analysis of ATP-binding sequences and structures that led to identification of sequence motifs and structural templates. For instance, the p-loop motif that interacts with ATP and its analogs was found in several protein families [4] and structural templates that interact with either adenosine or phosphates (the two chemical groups of ATP) were proposed [5,6]. However, these motifs/templates are usually confined to one or several protein families and cover only a small subset of the ATP-binding sites.

The large number of protein sequences which lack tertiary structure motivates development of computational tools for high-throughput sequence-based annotation of ATP-binding residues. At the same time, to the best of our knowledge, ATPint [7] is the only sequence-based predictor of the ATP-binding residues. We propose a novel method, named ATPsite, which aims to improve over the predictive quality of ATPint and other popular ways to annotate binging residues, including sequence alignment and conservation scoring. In contrast to the ATPint, which only takes PSSM profile and sequence descriptors as the inputs, the ATPsite uses a comprehensive set of relevant inputs. These inputs, which include PSSM profile, sequence descriptors, conservation scores, and predicted secondary structure, relative solvent accessibility (RSA), and dihedral angles, are encoded into a set of custom-designed features that are shown to improve the quality of the ATP-binding predictions.

## Methods

### Dataset

We extracted all complexes in PDB (as of February 2010) that include ATP. The maximal pairwise sequence identity of the resulting protein chains was reduced to 40% with CD-hit [8]. The remaining 227 chains that interact with ATP constitute the dataset used in this study. Similar to the annotation of DNA-binding residues and residues interacting with small ligands [9,10], a given residue is annotated as ATP-binding if at least one of its non-hydrogen atom is less than 3.9Å away from a non-hydrogen atom of the ATP molecule; our dataset includes 3393 ATP-binding residues and 80409 non-binding residues and is available at http://biomine.ece.ualberta.ca/ATPsite/.

### Evaluation criteria and test procedure

We use 5-fold cross validation to assess the predictions. We evaluated predictions at two levels: i) the binary value that defines whether a given residue does or does not bind to ATP; and ii) the real value that quantifies the probability of binding to ATP. The binary predictions were assessed using four measures

where *TP* (true positives) and *TN* (true negatives) are the counts of correctly predicted binding and non-binding residues, respectively, *FP* (false positives) are non-binding residues that were predicted as binding residues, and *FN* (false negatives) are binding residues that were predicted as non-binding residues. The Matthews correlation coefficient (MCC) ranges between -1 and 1 and it equals zero when all residues are predicted as binding or non-binding. Higher MCC value indicates better predictions.

The receiver operating characteristic (ROC) curve was used to examine the predicted probabilities. For each value of probability *p* achieved by a given method (between 0 and 1), the residues with probability ≥ *p* are set as the binding residue, and all other residues are set as the non-binding residue. Next, the TP-rate and the FP-rate are calculated and we use the area under the curve (AUC) to quantify the predictive quality.

We analyze statistical significance of the differences in the MCC and AUC values between predictions generated by ATPsite and the other methods. The MCC values are available for all methods while the AUC value cannot be calculated for an alignment-based predictor. These values are calculated per sequence (using the cross-validated predictions) for each method and we compare them using a paired Wilcoxon rank sum test at 0.01 significance. This non-parametric test is used since the per sequence MCC and AUC values do not follow normal distribution, as tested using Shapiro-Wilk test at the 0.05 significance.

### Architecture of the proposed predictor

**Figure 1:**
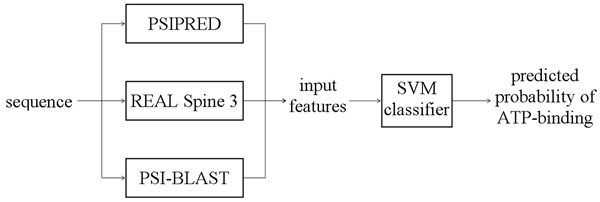
Architecture of the ATPsite predictor.

The architecture of the ATPsite predictor is shown in Figure 1. For a given protein sequence, PSIPRED [11] is used to predict the secondary structure, REAL Spine3 [12] predicts the RSA values and dihedral angles, and PSIBLAST [13] generates the PSSM profile. In addition to these inputs, other features such as conservation scores and amino acid (AA) groups are directly calculated from the sequence. Feature selection was performed to remove irrelevant and redundant features. The selected features are fed into a Support Vector Machine (SVM), which is implemented using LIBSVM [14], to generate probabilities of ATP-binding. Selection of this classifier is motivated by its successful use in the ATPint [7].

### Feature-based representation

The ATPsite utilizes both sequence and predicted structure descriptors as inputs. We utilize a sliding window of size 17 centered at the predicted residue to extract the input features. This window size was suggested in [7] to yield favorable predictive performance. The considered features include:

– *Predicted secondary structure* generated by PSIPRED [11]. We use probabilities of the 3 secondary structure states for each residue in the window.

– *Predicted relative solvent accessibility* generated by Real-SPINE3 [12]. We use the real values, which quantify the fraction of the surface area of a given residue that is accessible to the solvent, for the residues in the window.

– *Predicted dihedral angles* generated by Real-SPINE3 [12]. We utilize two real values, which represent *phi* (involving the backbone atoms C'-N-C^α^-C') and *psi* (involving the backbone atoms N-C^α^-C'-N) angles.

– *PSSM profile* generated by PSIBLAST [13] with default parameters. We normalize these inputs with 1/(1+2^-^*^x^*), where *x* is the raw value from the PSSM profile; this transformation is commonly used in secondary structure prediction. For a window centered at *R_i_* residue at *i*^th^ position, we calculate 17×20 features *f_i_*_+_*_k_*_,_*_j_* where *k*=−8, −7,…,7,8 is the index of the position in the window and *j*=1,2,…,20 is the index of the PSSM column. We averaged values on the left and right sides of the central residue *g_i_*_+_*_z_*_,_*_j_*=(*f_i_*_+_*_z_*_,_*_j_*+*f_i−z_*_,_*_j_*)/2 where *z*=0,1,…,8. As a result, the original 17×20 values are transformed to 9×20 values.

– *AA groups* including hydrophobic residues (Ala, Cys, Ile, Leu, Met and Val), negatively charged (Asp and Glu), positively charged (His, Lys, Arg) and carboxamide-containing AAs (Asn and Gln)*.*

– *Terminal indicator* is set to 1 for the first and last 3 residues in the sequence and 0 for the other positions.

– *Secondary structure segment indicator* for helix/ strand/ coil predictions from PSIPRED on both sides of the window is calculated. If 4 (3) consecutive residues on the left/right side of the window are predicted as helix (strand), we set the helix (strand) indicator as 1 for the left/right side. If both helix and strand indicators are 0, then the coil indicator is set as 1.

– *Residue conservation scores* are calculated based on the Shannon entropy (referred to as conservation A) and two other formulas proposed in [15,16] (named conservation B and C, respectively) which incorporate the background frequency of the amino acids.

– *Collocation of AA pairs*[*17*,*18*] is calculated for the residues in the window, which is motivated by results for membrane proteins where certain AA pairs are over-represented [19]. Similarly, several sequence motifs occur frequently at the ATP binding sites. To accommodate for mutations in these motifs, we use collocated AA pairs (pairs with gaps) to characterize these motifs. We only consider pairs formed between the central residue in the window and another residue up to 5 positions away. This results in 20×20×10=4000 frequencies (for 20 AA types and 10 positions; 5 on each side). The same as in the work for the membrane proteins [19], *p*-values that indicate the significance of the association between an AA pair and ATP-binding annotation are calculated. An AA pair with low *p*-value indicates a low probability that the association between this pair and ATP-binding is a coincidence. When analyzing 4000 randomly distributed variables, we expect to observe by chance one instance of a difference from expected value with significance *p* < 0.00025 (1/4000). We exclude the AA pairs with *p* ≥ 10^-6^, since based on the Engelman’s study [19] their association with ATP-binding event would be random.

We note that the terminal and secondary structure indicators, collocation of AA pairs, and the predicted secondary structure, relative solvent accessibility, and dihedral angles were never before used to predict the ATP-binding residues.

### Feature selection and parameterization

We use 5-fold cross validation to compute feature selection and evaluation. The dataset is randomly divided per-sequence into 5 folds, of which 4 are used for training and the one for testing; each of the 5 folds is used once as the test fold. This procedure assures that annotations of ATP binding from test folds are not used to train the predictive model. Biserial correlation is calculated between each of the features and the binary annotation of ATP-binding residues for each of the 5 training sets. The averaged, over the 5 training sets, correlation values were used to rank the features. We used a best first forward feature selection. Given a feature list *F*=[*f_i_*, *i*=1,2,…,*n*], sorted by the average correlation in the descendent order, and an empty list *S* consisting of selected features, in each round we add the top-ranked feature from *F* to *S* and run default SVM with linear kernel and complexity constant *C*=1 on the feature set *S* (using 5-fold cross validation). If addition of a given feature improves the average AUC value over the 5 test folds, this feature is retained in *S*; otherwise it is removed. We repeat that until *F* is empty.

The SVM is parameterized using the selected feature set *S* which includes 96 features. The features are summarized in Table 1. All parameterization steps maximize the average cross-validated AUC value. We considered polynomial and RBF kernels. For polynomial kernel, *C* is initially fixed at 1 and the degree of the polynomial is adjusted between 0.5 and 5. The degree that results in the highest average AUC is selected and next we adjust *C* with consecutive powers of 2 between 2^-3^ and 2^5^. Similarly for the RBF kernel, the *gamma* parameter is first optimized when *C* is fixed at 1, and next *C* is adjusted using the 2^-3^ and 2^5^ range. The threshold used to binarize the probabilities is set to maximize the MCC value of the predictions on the training folds. A residue is classified as ATP-binding if its predicted probability ≥ 0.182, which is the averaged threshold that maximizes the MCC of our method over the 5 training folds; otherwise it is categorized as non-binding.

**Table 1:** Summary list of the selected features.

Feature group	# of selected features
Predicted secondary structure	12
Predicted relative solvent accessibility	3
Predicted dihedral angles	6
PSSM profile	35
AA groups	11
Terminal indicator	0
Secondary structure segment indicator	1
Residue conservation scores	19
Collocation of AA pairs	9
Total	96

### Baseline predictors

ATPsite is compared with the existing ATPint as well as three baseline predictors that implement commonly used approaches to find binding residues:

– *Rate4site*[20] predicts functional sites by finding conserved residues. We first run PSI-Blast using the query sequence against the NCBI non-redundant database. For chains with at least 3 significant matches, we created alignments of the best 50 sequences (the default for Consurf [21], which is the web version of rate4site) using ClustalW [22] and we inputted them to rate4site. The rate4site generates conservation score for each residue and residues with lower scores (indicating a higher conservation) have higher probability to be binding residues. We use these scores to compute ROC curves and the threshold that maximizes the MCC value is used to binarize the conservation scores.

– *Sequence alignmentusing BLAST* identifies similar sequences or segments from a given annotated (with ATP-binding residues) dataset for a query sequence. This approach predicts the binding residues by using the ATP-binding annotations from the best aligned sequence. We execute the BLAST-based alignment between a query sequence and all other sequences (except the query sequence itself) in the benchmark dataset. The sequence with lowest E-value is selected as the template. The residues in the query sequence that were aligned with the binding residues on the template chain are predicted as the ATP-binding residues.

– *PSSM profile* is widely used in related sequence-based predictors, including the ATPint predictor [7]. To validate the effectiveness of the features proposed in this work, we build a simple predictor that uses SVM (which used the same parameters as the SVM in ATPsite) and takes PSSM profile as the input. This allows estimation of the improvements provided by the new features.

## Results

### Comparison with existing methods

**Figure 2:**
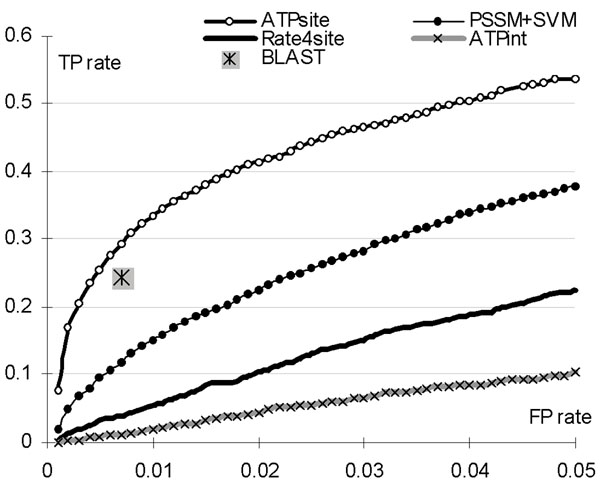
**ROC curves for ATPsite, ATPint, rate4site and the predictor based on PSSM with SVM classifier.** The FP-rate is constrained to [0, 0.05] range and the BLAST-based solution is shown using a single point that corresponds to the binary predictions. The full ROC curve can be found in the Supplementary Figure 1 in the online supplement at http://biomine.ece.ualberta.ca/ATPsite/.

The ATPsite predictor is compared with ATPint (we used the web server at http://www.imtech.res.in/raghava/atpint/) and the three baseline predictors based on the alignment, conservation scoring and evolutionary profiles, see Table 2. ATPsite is shown to outperform other methods. It improves the AUC and MCC by 0.03 and 0.07 when compared with the runner-up PSSM-based and BLAST-based predictors, respectively. These improvements are statistically significant with *p* < 0.01. ATPsite also achieved the highest (tied with BLAST) accuracy and the second best sensitivity and specificity. Since rate4site only considers residue conservation, it likely finds binding residues for other ligand types, catalytic residues, and key residues that are critical for protein folding. This explains the high sensitivity and low specificity, relative to the other methods, achieved by the rate4site. The alignment-based predictions have the best specificity and low sensitivity. This shows that some ATP-binding residues are in conserved sequence segments, but the sequence conservation can accommodate only for a fraction of the ATP-binding residues. The predictive quality of ATPint is lower than it was originally reported [7]. The authors of ATPint balanced the number of binding and non-binding residues in their study which likely lead to overestimation of the predictive quality when applied to full protein chains. Importantly, applications of the new features results in significant improvements as demonstrated by the differences between ATPsite and PSSM+SVM methods. The ROC curves are shown in Figure 2. The BLAST-based method does not provide probabilities and thus it is represented by a point that corresponds to the binary predictions. Figure 2 focuses on the FP rates < 0.05 since only about 4% of residues bind to ATP. The full ROC is given in the Supplementary Figure 1 in the supplement at http://biomine.ece.ualberta.ca/ATPsite/. The ATPsite achieves higher TP rates for the low FP rates consistently outperforming other solutions.

**Table 2:** Comparison between ATPsite, ATPint and three baseline predictors that use alignment (BLAST), conservation scoring (rate4site) and evolutionary profiles (PSSM+SVM). The “Significance” column reports statistical significance tests that compare paired per-sequence AUC and MCC between ATPsite and other methods; + indicates that ATPsite is significantly better at 0.01 level.

Predictor	Predicted probabilities		Predicted binary annotations				
	
	*AUC*	*Significance*	*SENS*	*SPEC*	*ACC*	*MCC*	*Significance*
ATPsite	**0.854**		0.361	0.988	**0.962**	**0.433**	
Rate4site	0.749	+	0.446	0.87	0.852	0.182	+
PSSM+SVM	0.824	+	0.354	0.957	0.933	0.27	+
BLAST	Not applicable		0.243	**0.993**	**0.962**	0.359	+
ATPint	0.627	**+**	**0.539**	0.651	0.648	0.078	**+**

### Effectiveness of individual input types

**Figure 3:**
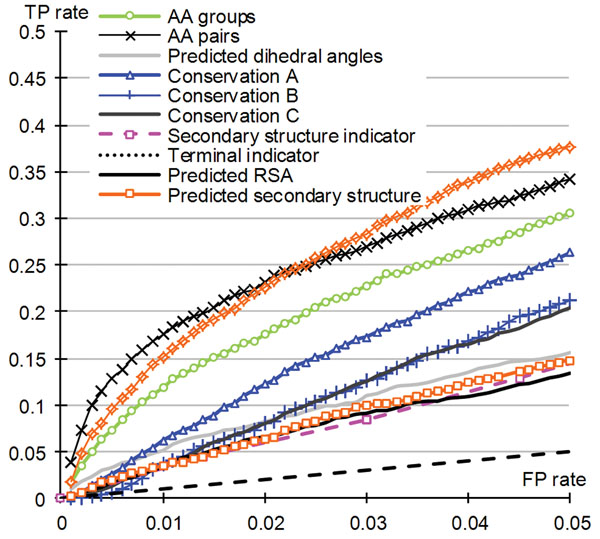
**ROC curves calculated based on predictions generated using individual input types.** The FP-rate is constrained to [0, 0.05]. The full ROC curve can be found in the Supplementary Figure 2 in the online supplement at http://biomine.ece.ualberta.ca/ATPsite/.

We assessed the ability of individual input types to predict the ATP-binding residues. We use the same SVM classifier but we utilize the selected features from one input source at the time, see Table 3. The corresponding ROC curves are shown in Figure 3 (for FP rates < 0.05) and in full in the Supplementary Figure 2 in the online supplement at http://biomine.ece.ualberta.ca/ATPsite/. Among the 11 feature groups, the PSSM profile achieves the highest AUC, followed by the AA groups and conservation scores. The 3 different types of scoring functions that we tried provide comparable predictive value. The AUC values for features based on predicted secondary structure and dihedral angles are above 0.7. Importantly, predictions obtained with these individual feature sets have substantially lower quality than predictions obtained with the combined set of features, which support the utility of our solution that uses and combines a set of novel inputs.

**Table 3:** Predictive quality achieved with individual input types; the inputs are sorted in the descending order using the AUC values.

Input type	# features	AUC	SENS	SPEC	ACC	MCC
PSSM profile	180	0.824	0.354	0.957	0.933	0.270
AA groups	36	0.785	0.390	0.924	0.902	0.218
Conservation B	17	0.745	0.470	0.860	0.844	0.181
Conservation A	17	0.744	0.427	0.892	0.873	0.194
Conservation C	17	0.741	0.564	0.792	0.783	0.170
Pred. secondary structure	51	0.732	0.651	0.720	0.717	0.161
Sec. str. segment indicator	6	0.706	0.656	0.683	0.682	0.143
Pred. dihedral angles	34	0.696	0.656	0.637	0.638	0.120
Collocation of AA pairs	153	0.671	0.182	0.990	0.957	0.261
Pred. solvent accessibility	17	0.628	0.194	0.918	0.889	0.079
Terminal indicator	1	0.517	0.989	0.045	0.084	0.033

### Consensus-based predictions

**Figure 4:**
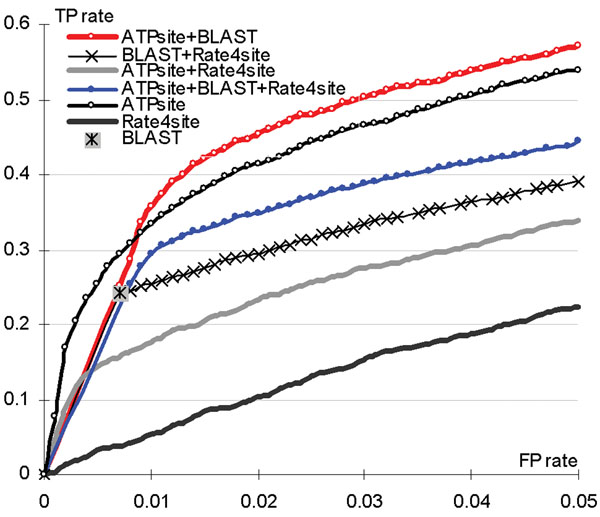
**ROC curves of the ensemble predictors.** The FP-rate is constrained to [0, 0.05]. The full ROC curve can be found in the Supplementary Figure 3 in the online supplement at http://biomine.ece.ualberta.ca/ATPsite/.

The machine-learning based ATPsite, alignment-based BLAST, and conservation-based rate4site utilize different and potentially orthogonal approaches, which motivates building consensus methods that would exploit this potential complementarity. We built 4 simple inclusive disjunction type ensembles of these 3 predictors (including 3 pairs of methods and all 3 methods together). For instance, the consensus of ATPsite and rate4site predicts a residue as ATP-binding if any of these two methods predicts this residue as binding; the corresponding probability equals to the maximal probabilities generated by the component predictors. The conservation scores of rate4site are linearly normalized to the [0, 1] range. We compare the ROC curves, AUC and MCC values, of the consensus and standalone predictors, see Figure 4 and Table 4 in the main text and the Supplementary Figure 3 in the online supplement at http://biomine.ece.ualberta.ca/ATPsite/. Only the consensus of ATPsite and BLAST improves the AUC and MCC values of the best standalone ATPsite. The consensus achieves AUC = 0.861 and MCC = 0.46 compared to 0.854 and 0.433 obtained with ATPsite, respectively. This suggests that the predictions from the ATPsite and the BLAST-based predictor are complementary. The likely reason why rate4site does not help is since ATPsite already utilizes conservation scores, although they are computed in a different way.

**Table 4:** Comparison between 4 ensemble predictors and their component predictors.

Method	AUC	MCC
ATPsite	0.854	0.433
BLAST	NA	0.359
Rate4site	0.749	0.182
ATPsite+BLAST	0.861	0.460
ATPsite+Rate4site	0.763	0.260
BLAST+Rate4site	0.779	0.234
ATPsite+BLAST+Rate4site	0.797	0.420

### The predicted probability implies confidence

**Figure 5:**
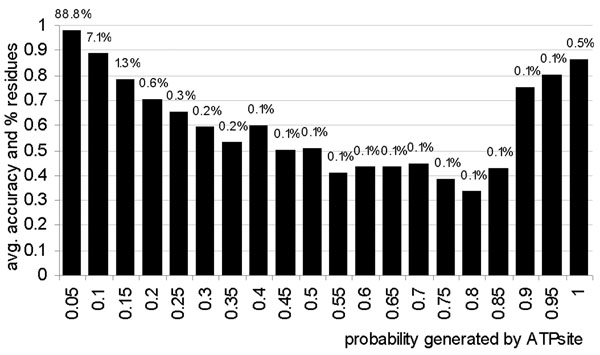
**The distribution of average accuracies (shown using bars) for residues binned into twenty 0.05 wide intervals based on their probability of ATP-binding predicted by ATPsite.** The percentage of the residues in each bin is shown above the bars.

We investigate whether the probabilities of the ATP-binding generated by ATPsite could be used as confidence scores for the binary predictions. We binned the residues into twenty 0.05 wide intervals based on their predicted probabilities and we computed the corresponding average accuracy and percentage of the residues in each of these bins, see Figure 5. For residues with high, >0.9, or low, <0.1, probabilities, the accuracies are higher than for the remaining residues. The averaged accuracy for 0.6% residues (including 14% of the ATP-binding residues) that are predicted with probability > 0.9 equals 0.85, and for the 94% of residues with probability < 0.1 it equals 0.98.

### Case study

**Figure 6:**
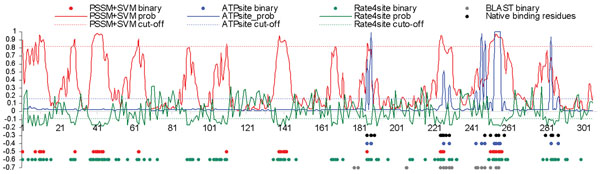
**Comparison of predictions for chain A of phosphofructokinase6 (PDB id 3CQD).** Solid lines at the top show the predicted probabilities for PSSM+SVM (in red), rate4site (green), and ATPsite (blue). The dashed lines denote cut-offs used to binarize the probabilities and the corresponding binary predictions are shown using dots (one dot per residue) at the bottom. Black dots denote native ATP-binding residues, and blue, red, green and gray denote predictions from ATPsite, PSSM+SVM, rate4site and BLAST, respectively.

We demonstrate predictions using chain A of phosphofructokinase6 (PDBid 3CQD). The predictive quality of the considered methods for this target is similar to their average quality on the entire dataset. The native ATP-binding residues, the binary predictions, and the probabilities predicted by ATPsite, rate4site, BLAST and PSSM+SVM methods are shown in Figure 6. The conservation scores from rate4site were divided by 10 to fit the Figure. The native ATP-binding residues are clustered into four segments, residue 185 to 189, 224 to 229, 248 to 258 and 280 to 287. ATPsite captured the four segments and correctly predicted 9 out of the 17 binding residues with 4 false positives (FPs). The PSSM profile-based predictor missed the last binding segment and successfully identified 7 out of the 17 binding residues with 23 FPs. The BLAST-based predictor missed two of the binding segments and correctly predicted 8 of the 17 binding residues with 7 FPs. The rate4site found the four segments and successfully identified 12 binding residue, however, it also produced 81 FPs.

## Conclusions

We developed a new method, ATPsite, for the sequence-based prediction of ATP-binding residues. Our predictor is empirically shown to outperform the existing approaches. These improvements are attributed to the usage of a novel and comprehensive set of input features, which include both sequence and predicted structural descriptors. We also found that a simple consensus of ATPsite with BLAST-based method leads to additional improvements. The consensus-based predictor achieves AUC = 0.861 and MCC = 0.46, which demonstrates that these predictions provide useful information for the high-throughput, sequence-based annotation of the ATP-binding residues.

## Abbreviations

SVM: Support Vector Machine; AUC: Area Under Curve; MCC: Matthews Correlation Coefficient; AA: Amino Acid; SENS: Sensitivity; SPEC: Specificity.

## Competing interests

The authors declare that they have no competing interests.

## Authors' contributions

KC and LK developed the prediction method and performed the experimental evaluation. MJM contributed to the collection of predictions by ATPint and KC processed the predictions by rate4site and BLAST. All authors contributed to writing the manuscript, and read and approved the final version.

## References

[B1] Berman HM Westbrook J Feng Z Gilliland G Bhat TN Weissig H Shindyalov IN Bourne PE The Protein Data Bank Nucleic Acids Res 2000 28 235 42 10.1093/nar/28.1.235 10592235 PMC102472

[B2] Maxwell A Lawson DM The ATP-binding site of type II topoisomerases as a target for antibacterial drugs Curr Top Med Chem 2003 3 283 303 10.2174/1568026033452500 12570764

[B3] Rock FL Mao W Yaremchuk A Tukalo M Crépin T Zhou H An antifungal agent inhibits an aminoacyl-tRNA synthetase by trapping tRNA in the editing site Science 2007 316 1759 1761 10.1126/science.1142189 17588934

[B4] Walker JE Saraste M Runswick MJ Gay NJ Distantly related sequences in the alpha- and beta-subunits of ATP synthase, myosin, kinases and other ATP-requiring enzymes and a common nucleotide binding fold EMBO J 1982 1 945 951 6329717 10.1002/j.1460-2075.1982.tb01276.x PMC553140

[B5] Moodie SL Mitchell JB Thornton JM Protein recognition of adenylate: an example of a fuzzy recognition template J Mol Biol 1996 263 486 500 10.1006/jmbi.1996.0591 8918603

[B6] Denessiouk KA Johnson MS When fold is not important: a common structural framework for adenine and AMP binding in 12 unrelated protein families Proteins 2000 38 310 26 10.1002/(SICI)1097-0134(20000215)38:3<310::AID-PROT7>3.0.CO;2-T 10713991

[B7] Chauhan JS Mishra NK Raghava GP Identification of ATP binding residues of a protein from its primary sequence BMC Bioinformatics 2009 10 434 10.1186/1471-2105-10-434 20021687 PMC2803200

[B8] Li W Godzik A Cd-hit: a fast program for clustering and comparing large sets of protein or nucleotide sequences Bioinformatics 2006 22 1658 1659 10.1093/bioinformatics/btl158 16731699

[B9] Luscombe NM Laskowski RA Thornton JM Amino acid-base interactions: a three-dimensional analysis of protein-DNA interactions at an atomic level Nucleic Acids Res 2001 29 2860 74 10.1093/nar/29.13.2860 11433033 PMC55782

[B10] Chen K Kurgan L Investigation of atomic level patterns in protein-small ligand interactions PLoS ONE 2009 4 4473 10.1371/journal.pone.0004473 PMC2637420 19221587

[B11] McGuffin LJ Bryson K Jones DT PSIPRED protein structure prediction server Bioinformatics 2000 16 404 5 10.1093/bioinformatics/16.4.404 10869041

[B12] Faraggi E Xue B Zhou Y Improving the prediction accuracy of residue solvent accessibility and real-value backbone torsion angles of proteins by guided-learning through a 2-layer neural network Proteins 2009 74 847 56 10.1002/prot.22193 18704931 PMC2635924

[B13] Altschul SF Madden TL Schäffer AA Zhang J Zhang Z Miller W Lipman DJ Gapped BLAST and PSI-BLAST: a new generation of protein database search programs Nucleic Acids Res 1997 25 3389 402 10.1093/nar/25.17.3389 9254694 PMC146917

[B14] Fan RE Chen PH Lin CJ Working set selection using second order information for training SVM J Mach Learn Res 2005 6 1889 918

[B15] Wang K Samudrala R Incorporating background frequency improves entropy-based residue conservation measures BMC Bioinformatics 2006 7 385 10.1186/1471-2105-7-385 16916457 PMC1562451

[B16] Capra JA Singh M Predicting functionally important residues from sequence conservation Bioinformatics 2007 23 1875 82 10.1093/bioinformatics/btm270 17519246

[B17] Chen K Kurgan L Ruan J Prediction of flexible/rigid regions from protein sequences using k-spaced amino acid pairs BMC Struct Biol 2007 7 25 10.1186/1472-6807-7-25 17437643 PMC1863424

[B18] Chen K Jiang Y Du L Kurgan L Prediction of integral membrane protein type by collocated hydrophobic amino acid pairs J Comput Chem 2009 30 163 72 10.1002/jcc.21053 18567007

[B19] Senes A Gerstein M Engelman DM Statistical analysis of amino acid patterns in transmembrane helices: the GxxxG motif occurs frequently and in association with beta-branched residues at neighboring positions J Mol Biol 2000 296 921 36 10.1006/jmbi.1999.3488 10677292

[B20] Pupko T Bell RE Mayrose I Glaser F Ben-Tal N Rate4Site: an algorithmic tool for the identification of functional regions in proteins by surface mapping of evolutionary determinants within their homologues Bioinformatics 2002 Suppl 1 S71 7 10.1093/bioinformatics/18.suppl_1.s71 12169533

[B21] Ashkenazy H Erez E Martz E Pupko T Ben-Tal N ConSurf 2010: calculating evolutionary conservation in sequence and structure of proteins and nucleic acids Nucleic Acids Res 2010 38 W529 33 10.1093/nar/gkq399 20478830 PMC2896094

[B22] Larkin MA Blackshields G Brown NP Chenna R McGettigan PA McWilliam H Clustal W and Clustal X version 2.0 Bioinformatics 2007 23 2947 2948 10.1093/bioinformatics/btm404 17846036

